# Factors Associated With the Work Intention of Hospital Workers’ in South Korea During the Early Stages of the COVID-19 Outbreak

**DOI:** 10.1017/dmp.2020.221

**Published:** 2020-06-25

**Authors:** Yeonhoon Jang, Myoungsoon You, Suyoung Lee, Wangjun Lee

**Affiliations:** Department of Public Health Science, Seoul National University Graduate School of Public Health, Seoul, South Korea; Department of Psychiatry, Myongji Hospital, Goyang, South Korea; Myongji Hospital, Goyang, South Korea

**Keywords:** COVID-19, disease outbreaks, hospital employees, intention to work, perceived threat

## Abstract

**Objective:**

This study aims to explore factors associated with the work intention of hospital workers in the early stages of the coronavirus disease (COVID-19) outbreak in South Korea.

**Methods:**

An online self-reported survey was conducted in a tertiary hospital. Respondents were asked to report their perceived threat and perceived risk of infection, evaluation of hospital response, demographics, and job-related factors. Descriptive statistics and multivariate regression analyses were performed.

**Results:**

A total of 441 employees participated in this study. Of respondents, 60% were willing to accept their work during an infectious disease outbreak and 12.5% were unwilling to accept the work. In addition, 8% of respondents reported that they had considered quitting their job, 54.4% reported that their job was dangerous, and 50.1% of respondents perceived the severity of COVID-19 as high. Perceived threat and effectiveness of hospital response were associated with hospital employees’ intention to work.

**Conclusions:**

Hospital workers are at the front line of the COVID-19 outbreak. This study highlighted hospital workers’ perceived effectiveness of organizational response to the outbreak, and perceived threats were found to be important factors for whether they continued to work or not in the fight against the outbreak.

The global spread of coronavirus disease (COVID-19) has led to a pandemic declaration by the World Health Organization, and countries around the world continue to fight the threat of this new virus.^1^ South Korea has also been coping with the threat of this pandemic; however, the experiences from this country may provide unique insight into the health care system during a public health emergency, since the COVID-19 outbreak occurred in South Korea earlier than in many other regions, including the United States and Europe.

Public health emergencies, such as the COVID-19 outbreak, cause an explosive increase in medical demand.^2,3^ Due to the surge in demand of medical services, hospitals must appropriately respond to the increase in the number of patients as well as infection control and intensive care facilities in hospitals. During a pandemic outbreak, hospital employees and health care workers provide essential elements for a hospital’s medical system to function appropriately,^4,5^ since they play a key role in not only safe and sustainable patient care, but also in the controlling of the outbreak.^6^ Several previous studies have reported outbreaks of infectious diseases: severe acute respiratory syndrome (SARS), avian influenza, and Middle Eastern respiratory syndrome coronavirus (MERS-CoV) can affect the willingness of health care providers to work,^7-10^ which could result in turnovers or health care workers’ intention to quit their jobs.^11,12^ Such negative influences on the work intention of hospital employees under the circumstances of an outbreak can lead to serious effects on maintaining sufficient health care services, which are necessary for both infected and noninfected patients. Therefore, it is necessary to manage the work intention of hospital workers during an infectious disease outbreak in order to adequately respond to a public health emergency.

The purpose of this study was to explore factors associated with the intention to work, such as willingness to accept work and consideration of quitting a job by hospital employees during the early stages of the COVID-19 outbreak. We also identified lessons and provide insight on the employment attitudes of health care workers who experienced the outbreak.

## METHODS

### Participants and Procedures

Quantitative data used in this study were collected from 1 hospital between February 6 and 12, 2020, which was the initial phase of the COVID-19 outbreak. This hospital runs government-designated negative pressure rooms for the infectious disease and received the third COVID-19-infected patient in South Korea. During the data collection, 2 more COVID-19 patients were admitted to this hospital, and 28 patients were confirmed with infection in South Korea. Since this hospital had provided health care for MERS-CoV patients in 2015, driving fear and stress among employees, a survey was constructed by employers to assess employees’ stress as a preemptive measure using an anonymous online questionnaire. The online questionnaire link was sent to all employees of the hospital via text messages. The retrospective analyses of this management survey were approved by the IRB (No. 2020-02-011-001), and there was acknowledgment of the necessity of further prospective surveys to assess the trend of intention and perception of hospital employees.

## MEASURES

Respondents completed an online self-reported questionnaire that included questions that covered several areas: (1) intention to work; (2) perceived risk of infection; (3) perceived threat during the COVID-19 outbreak; (4) evaluation of hospital response; (5) demographic characteristics; and (6) job data. The demographic characteristics of respondents included gender (1 = male, 2 = female), age, marital status (1 = married, 2 = single/bereavement/divorced), and presence of children (1 = yes, 2 = no). We also collected respondents’ information about job-related information, such as tenure, occupation (1 = nurse, 2 = physician, 3 = technician, 4 = administrator, 5 = other; classified as nurse, physician, or non-medical staff), employment status (1 = permanent, 2 = temporary), work experience during the MERS-CoV outbreak in 2015 (1 = yes, 2 = no), and type of COVID-19 task participation at present (1 = frontline worker, 2 = second-line worker, 3 = no participation) (Table 1).

**TABLE 1:** Demographic and Job Characteristics of Respondents (N = 441)

Characteristics	No.	%
Gender		
Male	105	23.8
Female	336	76.2
Age Groups	M = 36.78	SD = 10.2
20–29	131	29.7
30–39	149	33.8
40–49	104	23.6
50–59	46	10.4
> 60	11	2.5
Marital Status		
Married	195	44.2
Single/bereaved/divorced	246	55.8
Child		
Yes	172	39
No	269	61
Tenure	M = 8.51	SD = 6.9
1~2	88	20
3~4	85	19.3
5~7	81	18.4
8~11	72	16.3
> 11	115	26.1
Occupation		
Nurse	235	53.3
Physician	48	10.9
Non-medical staff	158	35.8
Employment Status		
Permanent	361	81.9
Temporary	80	18.1
MERS-CoV Work Experience		
Yes	99	22.4
No	342	77.6
Type of COVID-19 Work Participation^1^		
First-line	35	7.9
Second-line	109	24.7
No participation	297	67.3

1 First-line: healthcare for patients with COVID-19; second-line: temperature measurement, screening center.

The intention to work factor included 2 variables, which were the consideration of quitting (“I have thought about quitting my job due to the risk of COVID-19”) and willingness to work (“I accept my work given to me when infectious disease outbreaks occur, such as COVID-19”), rated on a 5-point Likert-type scale ranging from 1 to 5 with 1 = not at all and 5 = strong yes (Table 2).

**TABLE 2:** Working Intention of Hospital Employees During the COVID-19 Outbreak

Variable	Total	Nurse	Physician	Non-Medical	*P*-value	First-Line	Second-Line	None	*P*
Willingness to accept work					0.633				0.417
No	55 (12.5)	29 (12.3)	7 (14.6)	19 (12.0)		2 (5.7)	14 (12.8)	39 (13.1)	
Neither no nor yes	121 (27.4)	64 (27.2)	17 (35.4)	40 (25.3)		7 (20.0)	28 (25.7)	86 (29.0)	
Yes	265 (60.1)	142 (60.4)	24 (50.0)	99 (62.7)		26 (74.3)	67 (61.5)	172 (57.9)	
Consideration of quitting job					0.058				0.013
No	335 (76.0)	166 (70.6)	39 (81.2)	130 (82.3)		23 (65.7)	79 (72.5)	233 (78.5)	
Neither no nor yes	70 (15.9)	44 (18.7)	5 (10.4)	21 (13.3)		4 (11.4)	21 (19.3)	45 (15.2)	
Yes	36 (8.2)	25 (10.6)	4 (8.3)	7 (4.4)		8 (22.9)	9 (8.3)	19 (6.4)	

Respondents were asked to answer perceived risk and perceived threat of infection questions during the COVID-19 outbreak. Perceived risk of infection consisted of 2 items, perceived susceptibility (“What do you think is your possibility of a COVID-19 infection?”) and perceived severity of COVID-19 (“What do you think is the disease severity if you are infected with COVID-19?”), rated on a 5-point Likert-type scale ranging from 1 to 5 (1 = extremely low, 5 = extremely high). Perceived threat during the COVID-19 outbreak consisted of 8 questions. These items were adapted from those used in a previous study assessing the psychological impact of SARS on hospital employees in Taiwan,^9^ including perceived job risk (“I feel that my job is dangerous”), perceived stigma (“I feel that people around me avoid me because of my job”), perceived job stress (“I have felt more stress at work”), fear of infection (“I have a fear of COVID-19 infection”), little control (“I have little control over whether I get infected or not”), worry about transmission (“I worry that I will pass COVID-19 to others”), concern of others (“My family and friends are worried that they might get infected from me”), and thought of the possibility of death (“I have thought that I could die if I am infected with COVID-19”), rated on a 5-point Likert-type scale ranging from 1 to 5 (1 = strongly disagree, 5 = strongly agree).

The evaluation of hospital responses during the COVID-19 outbreak included “overall response of the hospital,” “hospital’s communication with employees,” and “response to staff safety and care of the hospital,” rated on a 5-point Likert-type scale ranging from 1 to 5 (1 = responded very poorly, 5 = responded very well) (Table 3). We used the evaluation of hospital response variable as the average value of the 3 items.

**TABLE 3:** Perceived Threat, Risk of Infection, and Evaluation of Hospital Response During the COVID-19 Outbreak

Variable^1^	Total	Nurse	Physician	Non-Medical	*P*-value	First-Line	Second-Line	None	*P*-value
Perceived Risk of Infection^2^									
Perceived susceptibility	31 (7.0)	20 (8.5)	2 (4.2)	9 (5.7)	0.004	6 (17.1)	9 (8.3)	16 (5.4)	0.126
Perceived severity	221 (50.1)	119 (50.6)	20 (41.7)	82 (51.9)	0.18	17 (48.6)	47 (43.1)	157 (52.9)	0.233
Perceived Threat^3^									
I feel my job is a dangerous one.	240 (54.4)	163 (69.4)	22 (45.8)	55 (34.8)	< 0.001	27 (77.1)	51 (46.8)	162 (54.5)	0.22
I think people avoid me because of my job.	153 (34.7)	89 (37.9)	9 (18.8)	55 (34.8)	0.12	15 (42.9)	33 (30.3)	105 (35.4)	0.354
I feel more stress at work.	153 (34.7)	97 (41.3)	15 (31.2)	41 (25.9)	0.029	19 (54.3)	44 (40.4)	90 (30.3)	0.03
I was afraid of infection to COVID-19.	167 (37.9)	96 (40.9)	19 (39.6)	52 (32.9)	0.577	15 (42.9)	30 (27.5)	122 (41.1)	0.028
I have little control over whether I get infected or not.	42 (9.5)	24 (10.2)	4 (8.3)	14 (8.9)	0.44	8 (22.9)	12 (11.0)	22 (7.4)	0.049
I’m worried I will pass COVID-19 to others.	154 (34.9)	82 (34.9)	16 (33.3)	56 (35.4)	0.512	16 (45.7)	32 (29.4)	106 (35.7)	0.283
My family and friends worried they might get infected through me.	165 (37.4)	84 (35.7)	11 (22.9)	70 (44.3)	0.033	16 (45.7)	39 (35.8)	110 (37.0)	0.346
I have thought that I could die if I get infected with the COVID-19.	59 (13.4)	36 (15.3)	4 (8.3)	19 (12.0)	0.697	5 (14.3)	15 (13.8)	39 (13.1)	0.96
Evaluation of Hospital Response	4.1(0.7)	4.3(0.7)	3.9(0.8)	4.1(0.6)	< 0.001	4.1(0.7)	4.1(0.7)	4.0(0.8)	0.686

1 Perceived Risk (perceived susceptibility and perceived severity), Perceived Threat: No. (%), Evaluation of Hospital Response: M (SD)

2 Sum of “high” and “extremely high.”

3 Sum of “agree” and “strongly agree.”

### Statistical Analysis

We conducted statistical analyses using R version 3.6.2 (R Foundation for Statistical Computing, Vienna, Austria). All results of quantitative variables were reported as either the number of responses (percentage %) or mean (M) and standard deviation (SD). We used the chi-square test and analysis of variance to identify the differences in responses by occupation as well as the COVID-19 task participation status. A multivariate linear regression analysis was performed to examine the effect of demographic factors, job-related factors, and COVID-19-related factors (ie, perceived risk of infection, fear of infection, fear of job, anxiety, perceived stigma, and organizational response) on the work intention of hospital employees (intention to quit, willingness to work). In addition, we used a multivariate linear regression model to estimate the associations among the noted demographic factors (ie, sex, age, marital status, presence of children), job-related factors (ie, tenure, occupation, employment status, MERS-CoV experience, type of COVID-19 work participation), individual factors, and organizational factors toward psychological well-being (psychological health and job stress during the COVID-19 outbreak).

## RESULTS

### Descriptive Statistics

A total of 441 hospital employees participated in this study, resulting in a response rate of 33%. Among respondents, 105 (23.3%) were men and 336 (76.7%) were women with a mean age of 37 years (M = 37.03, SD = 10.2; range = 22–76) (see Table 1). The 441 respondents in this study consisted of nurses (n = 235), physicians (n = 48), and non-medical staff (n = 158), the latter of which included technicians and administrators. In addition, 81.9% of hospital employees were permanent workers with a mean tenure of 8 years (M = 8.51, SD = 6.9; range = 1–31). Among the respondents, 44.2% had a spouse and 39% had children in the home. More than 2/3 (78.2%) of the respondents reported they did not experience MERS-CoV, 7.9% of participants were involved in the medical care of COVID-19 patients, and 24.7% of them participated in indirect work with these patients, such as temperature measurement.

### Intention to Work During the COVID-19 Outbreak

Of the respondents, 60.1% (n = 265) said they accepted their work when infectious diseases arose. Non-medical staff reported the highest proportion of acceptance of their work (62.7%), followed by nurses (60.4%) and physicians (50%) (*χ* ^2^ = 2.57, *P* = 0.633). In contrast, 12.5% of participants (n = 55) reported that they are unwilling to accept their job during an outbreak, with physicians being the highest (14.6%) followed by nurses (12.3%) and non-medical staff (12%). Those who worked as first-line workers for the COVID-19 outbreak represented the highest proportion of respondents (74.3%) who were willing to accept their work, followed by second-line workers (61.5%) and non-participation workers (57.9%) (*χ* ^2^ = 4.76, *P* = 0.417). There were no significant differences in the proportion of respondents who were willing to work among other demographics (ie, sex, age, marital status, or presence of children) or job-related factors (ie, tenure, job status, work experience during the MERS-CoV outbreak, and type of COVID-19 work participation).

Approximately 8% of respondents (n = 36) reported that they had thought about quitting their job during the COVID-19 outbreak. Nurses reported the highest rate (10.6%) of intention to quit among hospital employees and non-medical staff reported the lowest rate (*χ* ^2^ = 9.11, *P* = 0.058). In addition, 22% of respondents who were first-line workers for COVID-19 patient care reported that they considered quitting their job due to risk of infection, followed by those who had indirect participation (8.3%) or no participation (6.4%). These differences were statistically significant (*χ* ^2^ = 12.62, *P* = 0.013). No significant difference was observed in the proportion of respondents who considered quitting their job among demographic factors or job-related factors.

### Perceived Risk and Perceived Threat of Infection During the COVID-19 Outbreak

For the perceived risk of infection, only 7% of respondents reported that susceptibility of the COVID-19 is high. Nurses perceived the susceptibility as higher than other workers perceived, and there was a significant difference among professions; 50.1% of them answered that the severity of the virus is high, but no significant difference among occupations. The difference of perceived risk was not found among various types of health care workers.

For the perceived threat, the majority (54.4%) of respondents felt that their job was risky. Over 1/3 (34.7%) of participants reported that “people avoid me because of my job,” 34.7% “felt more stressed at work,” 37.9% “felt fear of the COVID-19 infection,” and 37.4% “worried passing COVID-19 to others.” The questions of “perceived job risk,” “perceived stress at work,” and “worry for family and friends” were statistically different among occupations. Nurses showed the highest concern for these questions. Workers who were in the first-line of COVID-19 patient care showed a significantly higher proportion of “perceived stress at work,” “being afraid of infection,” and “little control” than other types of workers.

### Evaluation of Hospital Response

Respondents also assessed the hospital response to the outbreak (ie, overall response, communication with employees, response to safety and care for employees during the COVID-19 outbreak). The average score of hospital responses was 4.1 (SD = 0.7) with nurses providing the highest score among occupations (M = 4.3, SD = 0.7). Physicians reported the lowest average score (M = 3.9, SD = 0.8). The differences in scores among occupations were statistically significant (F = 10.33, *P* < 0.001) but was not significant between types of work participation.

### Influencing Factors on the Work Intention of Hospital Employees During the Outbreak of COVID-19

Multivariate linear regression was performed to identify factors affecting the work intention of hospital workers during the COVID-19 outbreak (Table 4). The first-line work for COVID-19 patient care (β = 0.53, *P* = 0.004) and evaluation of hospital response (β = 0.19, *P* = 0.01) were positive and significant predictors, whereas “little control” (β = -0.25, *P* < 0.001) was a negative and significant predictor of willingness to accept work. Demographic factors (ie, sex, age, marital status, presence of children), tenure, experience with the MERS-CoV outbreak, and other perceived threats did not influence the willingness to work. These factors accounted for 9.1% of the variance in willingness to work (F [22, 418] = 3.01, adjusted R^2^ = 0.091, *P* < 0.001). Influencing factors on the intention to quit between independent variables were also assessed. “Nurse” (β = 0.29, *P* = 0.008), “feeling more stressed at work” (β = 0.21, *P* < 0.001), “little control” (β = 0.29, *P* < 0.001), and “thought of the possibility of death” (β = 0.21, *P* < 0.001) were positive and significant predictors for respondents to consider quitting their jobs. Evaluation of hospital response (β = -0.14, *P* = 0.017) was the only positive and significant predictor of the intention to quit. Demographic factors, MERS-CoV experience, type of participation for COVID-19 patient care, perceived susceptibility, perceived severity, and other perceived threats did not influence respondents’ consideration to quit. These factors accounted for 47.2% of the variance in the intention to quit (F [22, 418] = 18.89, adjusted R^2^ = 0.472, *P* < 0.001).

**TABLE 4:** Results of Multivariate Regression Analysis on Work Intention of Hospital Employee

	Willingness to Accept Work			Consideration of Quitting Job		
*Variables* ^1^	*β*	*se*	*p*	*β*	*se*	*p*
Constant	2.13	0.66	**0.001**	0.16	0.53	0.76
Sex: Female	-0.11	0.13	0.4	-0.11	0.1	0.29
Age	0.01	0.01	0.15	0.01	0.01	0.283
Marital Status: Married	0.13	0.16	0.418	0.03	0.13	0.806
Presence of Children: Yes	0.22	0.18	0.229	0.23	0.15	0.116
Tenure	0.04	0.05	0.414	-0.02	0.04	0.564
Occupation: Nurse	0.13	0.14	0.357	0.29	0.11	**0.008**
Occupation: Physician	-0.12	0.19	0.522	0.03	0.15	0.837
Job Status: Regular	0.02	0.15	0.911	-0.05	0.12	0.67
Working Experience in MERS-CoV: Yes	0.08	0.14	0.577	-0.06	0.11	0.569
Type of CoVID-19 work: First-line	0.53	0.19	**0.004**	0.07	0.15	0.63
Type of CoVID-19 work: Second-line	0.09	0.13	0.482	0.03	0.1	0.757
Perceived susceptibility	0.1	0.06	0.11	0.03	0.05	0.584
Perceived severity	0.02	0.05	0.692	-0.05	0.04	0.263
I felt my job is dangerous one.	-0.04	0.06	0.46	0	0.04	0.999
I think people avoid me because of my job.	-0.04	0.05	0.399	-0.02	0.04	0.662
I felt more stress at work.	0.04	0.05	0.408	0.21	0.04	**<0.001**
I was afraid of infection to COVID-19.	0.02	0.06	0.753	-0.02	0.05	0.642
I have little control over whether I get infected or not.	-0.25	0.06	**<0.001**	0.29	0.05	**<0.001**
I’m worried I will pass COVID-19 to others.	0.07	0.05	0.184	0.03	0.04	0.522
My family and friends worried they might get infected through me.	-0.05	0.05	0.301	0.05	0.04	0.223
I have thought that I could die if I get infected with the COVID-19.	0	0.05	0.98	0.21	0.04	**<0.001**
Evaluation of Hospital Response	0.19	0.07	**0.01**	-0.14	0.06	**0.017**
Adjusted R^2^	0.091			0.472		

1 Ref: Sex (Male), Marital Status (single/bereaved/divorced), Presence of Children (No), Occupation (Non-medical Staff), Job Status (Irregular), Working Experience in MERS-CoV (No), Type of COVID-19 work participation (No participation)

## DISCUSSION

The survey described in this study was conducted during the initial phase of the COVID-19 outbreak in South Korea when there were 28 confirmed cases in the country and 3 patients were admitted to this hospital. The study revealed that approximately 60% of hospital workers were willing to accept their work duties, which is similar to previous results reported on the willingness of hospital workers to work during an infectious disease outbreak.^7-10^ However, 8.1% of respondents thought about quitting their jobs due to the risk of COVID-19 infection, which is lower than that reported in a previous study, where 25.9% had considered leaving their jobs.^12^ The results could be explained by the survey conducted in the initial phases of the COVID-19 outbreak and respondents’ perceived susceptibility of infection as low. However, we also found that respondents who considered quitting their job in the early stages of the outbreak also existed. Thus, the analysis of intention to work will be important for the preemptive response in the outbreak of infectious disease, and further studies are needed to monitor changes in hospital employees’ willingness to work and their consideration of quitting their jobs as the intensity of the outbreak changes.

The results of our study showed that, although the proportion of respondents who considered quitting their jobs was higher among nurses and first-line workers than other employees, there was no difference in the willingness to work based on task participation. This could be associated with the nature of hospital workers’ roles and perceived obligation to respond to an emergency situation.^13,14^ A previous study reported similar results showing that health care workers’ perceived responsibility was associated with the willingness to work during an infectious disease outbreak.^15^

The multivariate regression analyses indicated that respondents who had little control over their anxiety were unwilling to accept their tasks, while factors such as “feeling more stress” and “having a hard time controlling fear” were positively associated with the consideration of quitting their jobs during the outbreak. This is in a similar context of a previous study, which reported that health care workers felt that they had little control of anxiety due to uncertainty and lack of information during the initial phase of the outbreak,^5,9,16^ resulting in more stress. Some variables were adjusted for other variables, leading to statistically non-significant results in our analyses. For instance, “perceived job risk” could have been replaced by “possibility of death” for consideration of quitting their jobs. This could be explained by the notion that the former factor focuses on risk of infection, whereas the latter focuses on the risk of a fatal consequence. Similarly, anxiety of self would be more important for consideration of quitting their jobs and willingness to accept their work than perceived stigma and worry for others.^17^ This could be interpreted as personal fear having more impact than concern for their relationship with other people.

It is worth noting in our study that the evaluation of the hospital response was significantly related to the intention to work. Multivariate regression analyses showed that respondents who positively assessed their hospital’s response were more willing to accept their work and have less consideration of quitting during the COVID-19 outbreak. This indicates that the role of the hospital, such as communication with employees, response to safety, and care of staff, is quite important during an infectious disease outbreak. Several previous studies have reported that health care workers recognize hospital response and support as important factors. The intention to work and willingness to work increase when hospitals provide personal protective equipment (PPE)^18-20^ and accurate information appropriately.^10,21^

Several recommendations arise from this study that may prevent a negative attitude toward the work intention of hospital employees during the initial phase of an infectious disease outbreak. First, the response of the hospital should be emphasized. Hospitals need to supervise and monitor the safety of employees by providing appropriate PPE and mental health support during an outbreak. Previous studies have reported that fear and anxiety are associated with a willingness to work,^8,22^ and the provision of infection control measures would positively affect the work intention of hospital workers.^13,18,19,21^ In particular, hospitals need to consider the types of tasks that workers conduct during emergency situations. The results of our study showed that perceptions of stress, anxiety, and fear of infection in first-line workers are higher than those of other workers during the COVID-19 outbreak. Thus, frontline workers should receive priority for support from the hospital, not only with provisions for infection control measures, but also intervention for psychological health (eg, psychological brochures, psychotherapy, and counseling),^23^ which would be helpful for alleviating an unwillingness to work. Providing sufficient training and education is another important factor that affects the work intention of hospital employees. Continuous education and training should be provided to adequately deal with public health emergencies in the future.^24,25^ The sharing of pandemic preparedness planning, information, and communication between the employer and employees is important not only for increasing the willingness to work,^21^ but also for improving the trust in employers.^10,26^ A reciprocal relationship between the employer and employee should also be constructed.

It is also necessary for organizations to ensure the safety of hospital workers’ families. In previous studies, the intention to work was highly associated with the safety of health care workers’ families.^20,21,27,28^ Hospital employees working during an outbreak are often concerned about their families due to possibility of infection, which becomes a major barrier for the willingness to work. The willingness to work has been shown to increase when PPE is made available for workers’ families.^21^ Thus, support for employees’ families would be effective for mitigating the concern of workers. Finally, compensation plays a key role in maintaining a sufficient health care workforce during the COVID-19 outbreak. Health care personnel reported that a positive incentive to keep working during the pandemic was related to special compensation, such as increased wages, additional vacation, and recognition,^12,29^ whereas the intention to work did not increase when they were threatened by employers of losing their license.^18^ Therefore, hospitals and governments should develop and implement guidelines and policies to incentivize hospital personnel to remain working during infectious disease outbreaks.

Our study had several limitations. First, we only reported on the work intention of hospital employees during the early stages of the COVID-19 outbreak. The severity of the situation in South Korea was relatively less serious than other areas of the world, which indicates that there is a possibility that the perceived threat and job attitude of health care workers were underestimated. Second, the survey was not conducted with structured questionnaires, which made it impossible to compare the results to previous studies. There were a few scales that were substituted with several variables using single items. In addition, the variables of health care workers’ perception of the provision of infection control measures were absent, such as N-95 masks, gowns, and gloves, which were frequently used in previous studies. Third, the respondents of our study were limited to a single tertiary hospital, which makes it difficult to compare to health care workers in other settings, such as primary health care. In addition, the number of respondents who were physicians was limited, so it was difficult to compare the results with other occupations. Finally, due to the exploratory nature of this study, theoretical models were absent. The purpose of our study was to determine the perception and attitude of hospital employees, rather than testing theoretical hypotheses. Therefore, it is difficult to postulate a systematic explanation for the result of this study.

## CONCLUSION

This exploratory study was conducted to determine tertiary hospital workers’ intention to work during the COVID-19 outbreak. Hospital workers are at the front line of the COVID-19 outbreak. This study emphasized that hospital workers’ perceived effectiveness of organizational responses to the outbreak as well as their perceived threat are important factors determining whether they continue to work or not in the fight against this infectious disease. We expect that further studies will be conducted to monitor changes in hospital employees’ intention to work during the COVID-19 outbreak.

## References

[1] Bao Y , Sun Y , Meng S , et al. 2019-nCoV epidemic: address mental health care to empower society. Lancet. 2020;395(10224):e37-e38. 32043982 10.1016/S0140-6736(20)30309-3 PMC7133594

[2] Perry RW . What is a disaster? In: Rodriguez H , Quarantelli EL , Dynes R , et al., eds. Handbook of Disaster Research. Handbooks of Sociology and Social Research. New York: Springer; 2007.

[3] Chaffee M . Willingness of health care personnel to work in a disaster: an integrative review of the literature. Disaster Med Public Health Prep. 2009;3(1):42-56. 19293743 10.1097/DMP.0b013e31818e8934

[4] Kaji A , Koenig KL , Bey T . Surge capacity for healthcare systems: a conceptual framework. Acad Emerg Med. 2006;13(11):1157-1159. 16968688 10.1197/j.aem.2006.06.032

[5] Charney RL , Rebmann T , Flood RG . Hospital employee willingness to work during earthquakes versus pandemics. J Emerg Med. 2015;49(5):665-674. 26371972 10.1016/j.jemermed.2015.07.030

[6] Chang D , Xu H , Rebaza A , et al. Protecting health-care workers from subclinical coronavirus infection. Lancet Respir Med. 2020;8(3):e13. 32061333 10.1016/S2213-2600(20)30066-7 PMC7128440

[7] Koh D , Lim MK , Chia SE , et al. Risk perception and impact of severe acute respiratory syndrome (SARS) on work and personal lives of healthcare workers in Singapore: what can we learn? Med Care. 2005;43(7):676-682. 15970782 10.1097/01.mlr.0000167181.36730.cc

[8] Wong EL , Kung K , Cheung AW , et al. Will the community nurse continue to function during H1N1 influenza pandemic? – a cross-sectional study of Hong Kong community nurses. BMC Health Serv Res. 2010;10. doi: 10.1186/1472-6963-10-107. PMC2907760 20433691

[9] Chong MY , Wang WC , Hsieh WC , et al. Psychological impact of severe acute respiratory syndrome on health workers in a tertiary hospital. Br J Psychiatry. 2004;185:127-133. 15286063 10.1192/bjp.185.2.127

[10] Gershon RR , Magda LA , Qureshi KA , et al. Factors associated with the ability and willingness of essential workers to report to duty during a pandemic. J Occup Environ Med. 2010;52(10):995-1003. 20881624 10.1097/JOM.0b013e3181f43872

[11] Jung H , Jung SY , Lee MH , Kim MS . Assessing the presence of post-traumatic stress and turnover intention among nurses post–Middle East respiratory syndrome outbreak: the importance of supervisor support. Workplace Health Saf. 2020;epub, doi: 10.1177/2165079919897693. PMC7201205 32146875

[12] Shiao JS , Koh D , Lo LH , et al. Factors predicting nurses’ consideration of leaving their job during the SARS outbreak. Nurs Ethics. 2007;14(1):5-17. 17334166 10.1177/0969733007071350

[13] Irvin CB , Cindrich L , Patterson W , Southall A . Survey of hospital healthcare personnel response during a potential avian influenza pandemic: will they come to work? Prehosp Disaster Med. 2008;23(4):328-335. 18935947 10.1017/s1049023x00005963

[14] Damery S , Draper H , Wilson S , et al. Healthcare workers’ perceptions of the duty to work during an influenza pandemic. J Med Ethics. 2010;36(1):12-18. 20026687 10.1136/jme.2009.032821

[15] Cone DC , Cummings BA . Hospital disaster staffing: if you call, will they come? Am J Disaster Med. 2006;1(1):28-36. 18274041

[16] Maunder R , Hunter J , Vincent L , et al. The immediate psychological and occupational impact of the 2003 SARS outbreak in a teaching hospital. CMAJ. 2003;168(10):1245-1251. 12743065 PMC154178

[17] Tzeng HM , Yin CY . Nurses’ fears and professional obligations concerning possible human-to-human avian flu. Nurs Ethics. 2006;13(5):455-470. 16961111 10.1191/0969733006nej893oa

[18] Martin SD . Nurses’ ability and willingness to work during pandemic flu. J Nurs Manag. 2011;19(1):98-108. 21223410 10.1111/j.1365-2834.2010.01190.x

[19] Mackler N , Wilkerson W , Cinti S . Will first-responders show up for work during a pandemic? Lessons from a smallpox vaccination survey of paramedics. Disaster Manag Response. 2007;5(2):45-48. 17517362 10.1016/j.dmr.2007.02.002 PMC7110586

[20] Seale H , Leask J , Po K , MacIntyre CR . “Will they just pack up and leave?” – attitudes and intended behaviour of hospital health care workers during an influenza pandemic. BMC Health Serv Res. 2009;9:30. 19216792 10.1186/1472-6963-9-30 PMC2661074

[21] Garrett AL , Park YS , Redlener I . Mitigating absenteeism in hospital workers during a pandemic. Disaster Med Public Health Prep. 2009;3(Suppl 2):S141-S147. 19952885 10.1097/DMP.0b013e3181c12959

[22] Tzeng HM , Yin CY . A crisis: fear toward a possible H5n1 pandemic. J Nurs Care Qual. 2008;23(2):177-183. 18344785 10.1097/01.NCQ.0000313768.17514.a3

[23] Kang L , Ma S , Chen M , et al. Impact on mental health and perceptions of psychological care among medical and nursing staff in Wuhan during the 2019 novel coronavirus disease outbreak: a cross-sectional study. Brain Behav Immun. 2020;epub, doi: 10.1016/j.bbi.2020.03.028. PMC7118532 32240764

[24] Liu Q , Luo D , Haase JE , et al. The experiences of health-care providers during the COVID-19 crisis in China: a qualitative study. Lancet Glob Health. 2020;8(6):e790-e798. 32573443 10.1016/S2214-109X(20)30204-7 PMC7190296

[25] Shi Y , Wang J , Yang Y , et al. Knowledge and attitudes of medical staff in Chinese psychiatric hospitals regarding COVID-19. Brain Behav Immun Health. 2020;4:100064. 32289123 10.1016/j.bbih.2020.100064 PMC7138160

[26] Ives J , Greenfield S , Parry JM , et al. Healthcare workers’ attitudes to working during pandemic influenza: a qualitative study. BMC Public Health. 2009;9(1):56. 19216738 10.1186/1471-2458-9-56 PMC2654560

[27] Qureshi K , Gershon RR , Sherman MF , et al. Health care workers’ ability and willingness to report to duty during catastrophic disasters. J Urban Health. 2005;82(3):378-388. 16000654 10.1093/jurban/jti086 PMC3456052

[28] Nickell LA , Crighton EJ , Tracy CS , et al. Psychosocial effects of SARS on hospital staff: survey of a large tertiary care institution. CMAJ. 2004;170(5):793-798. 14993174 10.1503/cmaj.1031077 PMC343853

[29] Khalid I , Khalid TJ , Qabajah MR , et al. Healthcare workers emotions, perceived stressors and coping strategies during a MERS-CoV outbreak. Clin Med Res. 2016;14(1):7-14. 26847480 10.3121/cmr.2016.1303 PMC4851451

